# Disparities in screening participation and stage at diagnosis for breast and bowel cancers among individuals with pre-existing mental health disorders: A population-based study

**DOI:** 10.1371/journal.pone.0361617

**Published:** 2026-10-27

**Authors:** Yueh-Hsin Wang, Ajay Aggarwal, Elizabeth A. Davies

**Affiliations:** 1 Cancer Epidemiology and Cancer Services Research, Centre for Cancer Society & Public Health, Comprehensive Cancer Centre, School of Cancer & Pharmaceutical Sciences, Faculty of Life Sciences & Medicine, King’s College London, London, United Kingdom; 2 Institute of Cancer Policy, Comprehensive Cancer Centre, School of Cancer & Pharmaceutical Sciences, Faculty of Life Sciences & Medicine, King’s College London, London, United Kingdom; 3 Department of Health Services Research and Policy, Faculty of Public Health and Policy, London School of Hygiene and Tropical Medicine, London, United Kingdom; Taipei Medical University, TAIWAN

## Abstract

People with pre-existing mental health disorders (PMHD) often have lower cancer screening uptake and later-stage diagnoses. It remains unclear whether these disparities persist within highly accessible universal healthcare systems without strict gatekeeping. We simultaneously investigated the associations between PMHD and both screening participation and stage at diagnosis of breast and bowel cancers within the Taiwan health service. Using nationwide National Health Insurance and Cancer Registry data covering over 99% of the population, we compared outcomes between individuals with and without PMHD. Propensity score matching was applied for the screening cohort. Adjusted odds ratios were estimated using logistic regression, with subgroup analyses for severe PMHD. Overall, PMHD was associated with higher screening participation (adjusted odds ratios [aOR]: breast = 1.04, bowel = 1.12) and reduced odds of metastatic disease at diagnosis (breast = 0.72, bowel = 0.93), although the magnitude of these associations was modest, particularly for bowel cancer. Conversely, individuals with severe PMHD had significantly lower odds of screening (breast = 0.81, bowel = 0.88) despite high outpatient use. Furthermore, while the severe group retained reduced odds of metastatic breast cancer at diagnosis (aOR = 0.79), they showed no staging advantage for bowel cancer. While Taiwan’s highly accessible universal healthcare system may contribute to a surveillance effect benefiting the general PMHD population, significant disparities persist for individuals with severe illnesses. Targeted interventions, procedural simplifications for self-administered tests, and multidisciplinary person-centred care models, may be needed to ensure health equity.

## Introduction

Cancer and mental health disorders are both major global health concerns, showing significant co-morbidity. Individuals with pre-existing mental health disorders (PMHD) often experience significantly poorer cancer survival. While disparities in cancer outcomes are influenced by multiple factors such as treatment and comorbidities, stage at diagnosis is a critical determinant of prognosis [1]. Evidence indicates that PMHD is generally associated with advanced stage at diagnosis [2,3] and a prolonged interval from symptomatic consultation to diagnosis [4]. However, this association is not uniform. Some studies indicate no significant disparities, suggesting that outcomes may vary by mental health condition or healthcare system.

Screening is widely adopted as a primary approach to facilitate the earlier detection of cancers, but as a multi-step pathway, it may be less often undertaken by individuals with PMHD. While some studies show no significant disparities [5], evidence from other studies consistently indicates reduced uptake across diverse breast and bowel screening modalities [6–13]. These disparities stem from a combination of individual and systemic barriers. Individually, social, emotional, or cognitive disturbances can impair a patient’s ability to recognise physical symptoms or navigate complex healthcare pathways [2,5,6,14–16]. The impact of these difficulties is often exacerbated by the specific screening modality required, leading to issues such as poorer adherence to colonoscopy protocols and incomplete procedures [6,11,13,14,16]. Systemic barriers, including financial constraints and fragmented healthcare services, can lead to prolonged referral intervals and missed screening opportunities, compounding the risk of a late diagnosis [4,5,14–18]. Furthermore, the lack of trauma-informed care and negative experiences of compulsory treatment from mental health services impact trust in health services, resulting in lower cancer screening participation [5,19–22].

However, it remains unclear whether these disparities in screening participation and stage at diagnosis persist in healthcare systems with universal coverage and high accessibility. Most previous studies have reported from countries with healthcare gatekeeping or non-universal coverage healthcare systems, where systemic barriers may be the main challenges in cancer care among people with PMHD. In contrast, a system with high medical use theoretically provides a surveillance effect, providing frequent healthcare engagements for people with mental illness, which may increase both screening participation and opportunistic cancer detection [5,9,10]. Nevertheless, it is uncertain whether a high frequency of medical visits can compensate for barriers that impact people, such as trust in the system or a lack of trauma-informed care, particularly when screening or diagnostic procedures require supported and targeted outreach. Taiwan, with its National Health Insurance (NHI), offers a unique context to investigate the persistence of these disparities within a healthcare system characterised by universal coverage and high accessibility.

The Taiwanese NHI, implemented in 1995, has achieved 99% population enrolment and has service contracts with 92.44% of all health facilities [23]. Given its high healthcare accessibility and the absence of referral restrictions, the average per capita number of outpatient visits has reached 13.12 per year [23], providing a unique context with minimal systemic barriers to healthcare access. In addition, breast and bowel cancer screening programmes have been subsidised for high-risk populations for over two decades to address the high prevalence of these cancers in Taiwan. Moreover, population-based data on cancer registries, screening participation, and healthcare use have been comprehensively recorded in national databases for more than 20 years, enabling robust longitudinal analysis of the cancer care pathway for individuals with PMHD. Consequently, this study employs a nationwide population-based design to compare (1) cancer stage at diagnosis and (2) screening attendance between individuals with and without PMHD for breast and bowel cancers. Furthermore, we examine these disparities by focusing on individuals with severe PMHD to determine whether the severity of mental illness remains a persistent barrier to cancer care, even within a universal healthcare system characterised by high accessibility.

## Materials and methods

### Data sources and record linkage

This retrospective cohort study linked data from the National Health Insurance Research Database (NHIRD; 2016–2021) and Taiwan Cancer Registry (TCR; 2002–2021) using encrypted unique identifiers. NHIRD, covering 99.9% of the Taiwanese population, provides longitudinal claims data. It includes outpatient and emergency department visits, hospital admissions, and prescription records, which were used to identify pre-existing mental health disorders, comorbidities, and screening attendance. TCR provides histologically confirmed cancer diagnoses and detailed tumour staging information based on the American Joint Committee on Cancer (AJCC) system. The AJCC staging system 7th Edition was used for people newly diagnosed with cancer from 2016 to 2017, and the 8th Edition was used from 2018 to 2021.

### Study design and population

To address the study aims, we established four distinct cohorts stratified by cancer type (breast and bowel) and study outcome (stage at diagnosis and screening attendance). These cohorts were organised under two primary analytical frameworks. Firstly, for the stage at diagnosis (S1–S3 Figs), individuals newly diagnosed with invasive breast cancer (International Classification of Diseases for Oncology, 3rd Edition [ICD-O-3]: C50) or bowel cancer (ICD-O-3: C18–C21) between 2016 and 2021 were included. Participants were excluded if they were (1) aged <18 years, (2) male (for breast cancer analysis), (3) diagnosed with carcinoma in situ (CIS), or (4) missing staging or demographic information (sex, income, or urbanisation). Secondly, for cancer screening attendance (S4–S6 Figs), the study population comprised individuals eligible for free breast or bowel cancer screening in 2019, including women aged 45–69 years for mammography and people aged 50–74 years for the Faecal Immunochemical Test (FIT). Individuals were excluded if they (1) were diagnosed with breast or bowel cancers prior to 2019, (2) had died during the observation period (2019–2021), (3) had a new breast or bowel cancer diagnosis during the observation, or (4) in the control group but diagnosed with a mental health illness during the study period, so ensuring a clean comparison. For the staging analysis, all eligible patients were included without matching to maximise statistical power, and potential confounders were adjusted through multivariable regressions. In contrast, the screening analysis compared individuals with PMHD against the general population. Given the potential substantial baseline disparities, a 1:5 propensity score matching using a greedy nearest-neighbour algorithm was applied to mitigate selection bias, matching individuals with PMHD to those without PMHD based on age, monthly income, and sex (for the bowel cohort only).

### Definition of PMHD

PMHD was identified from diagnostic records over the 3-year period preceding the index date (1 January 2019 for the screening cohort and the date of cancer diagnosis for the staging cohort). Mental disorders were defined using codes from the International Classification of Diseases, Ninth Revision, Clinical Modification (ICD-9-CM), and International Classification of Diseases, Tenth Revision (ICD-10) (complete list of codes provided in S1 Table). These codes were identified from inpatient, emergency, and outpatient claims. Disorders of organic or neurocognitive origin, such as dementia and delirium, were excluded. To ensure diagnostic validity and to exclude transient or tentative diagnoses, individuals were classified as having PMHD if they had at least one outpatient, emergency department visit, or inpatient admission with a primary diagnosis accompanied by the prescription of psychotropic medications, including antipsychotics, anxiolytics, hypnotics/sedatives, or antidepressants. Conversely, to ensure a distinct separation from those with PMHD, the comparison group consisted of individuals with no mental disorder diagnoses during the lookback and study observation period. Moreover, to investigate disparities in stage at diagnosis and screening participation for people with severe disorders, those people with a history of hospitalisation or emergency department visits for mental health conditions were identified from the PMHD group and categorised as having severe PMHD for sub-analyses.

### Covariates

To account for potential confounding, statistical models were adjusted for personal and clinical characteristics. Regarding personal characteristics, age was calculated from the index date (1 January 2019 for screening; date of diagnosis for staging) and categorised into 5-year or 10-year age groups. Socioeconomic status was proxied by monthly insurance premium levels, which correlate with income tax brackets in Taiwan. Urbanisation was assessed using Liu’s seven-category system, ranging from 1 (most urbanised) to 7 (least urbanised) [24]. Clinical factors, including comorbidities, were measured using the Charlson Comorbidity Index (CCI) [25]. Scores were calculated based on diagnostic codes (ICD-9-CM/ICD-10-CM) recorded in inpatient and outpatient claims within the 2-year lookback period preceding the index date, and were classified into 0, 1, and ≥2 categories. Healthcare use was defined as the average annual number of outpatient visits calculated over the 3-year lookback period preceding the index date. This variable captures the individual’s longitudinal intensity of interaction with the healthcare system. Participants were categorised into three groups based on the distribution of these annual averages across the study population: < Q1 (low use), Q1–Q3 (moderate use), and >Q3 (high use). Cancer history, defined as a diagnosis of any cancer between 2002 and the index date, was obtained from the Taiwan Cancer Registry.

### Outcome measures

Outcomes were assessed across two domains, including stage at diagnosis and screening attendance. Cancer stage was obtained from TCR based on AJCC TNM staging. In the primary analysis, cancer stage was categorised as non-metastatic disease (Stages I–III) versus (vs) metastatic disease (Stage IV), reflecting our aim to distinguish cancers without distant metastasis from those with distant metastasis at diagnosis. Although Stage III disease is generally considered locally advanced rather than early-stage disease, it remains non-metastatic. As a sensitivity analysis, cancer stage was additionally categorised as Stage I–II versus Stage III–IV to assess the robustness of the findings.

Regarding screening attendance, a 3-year observation period (2019–2021) was selected to fully capture compliance with the national biennial (2-year) screening guidelines, allowing for minor deviations in attendance timing. Breast cancer screening was defined as the uptake of at least one government-funded mammography, whereas bowel cancer screening was defined as the uptake of at least one FIT. While the bowel screening programme includes follow-up colonoscopy for positive cases, this study focused specifically on the initial uptake of FIT as the primary measure of screening accessibility and intent.

### Statistical analysis

Continuous variables (means ± standard deviation [SD]) were compared using Student’s t-tests, and categorical variables (frequencies/percentages) using Chi-square tests. Missing data were excluded from the analysis. The staging analysis included all eligible newly diagnosed patients using multivariable adjustment, whereas the screening analysis used a 1:5 PSM procedure to mitigate selection bias. PSM was performed using a greedy nearest-neighbour algorithm without replacement, with a calliper width of 0.2 of the pooled standard deviation of the logit of the propensity score. Each patient with PMHD was matched to five controls (1:5 ratio). To assess covariate balance, standardised mean differences (SMDs) were calculated before and after matching (S2 Table), with an SMD < 0.1 indicating adequate balance [26,27]. Following these preparatory steps, multivariable logistic regression models were constructed to estimate adjusted odds ratios (aORs) and 95% confidence intervals (CIs). The variance inflation factor (VIF) was used to evaluate multicollinearity among covariates in the multivariable logistic regression models. VIF values of 2.5 or greater are considered indicative of considerable collinearity [28].

Separate models, adjusted for potential confounders, were developed to address the two objectives. For the cancer staging analysis, the odds of metastatic disease at diagnosis (Stage IV vs Stages I–III) were evaluated using multivariable logistic regression, with adjustments for age, sex (for bowel cancer), monthly income, urbanisation, CCI score, and history of cancer. Furthermore, a sensitivity analysis was conducted using the conventional Stages I–II versus Stages III–IV classification, with Stages III–IV representing locally advanced or metastatic disease, to evaluate the robustness of the study findings. Similarly, for the screening attendance analysis, despite the initial matching, models were further adjusted for age, sex (for bowel cancer), and monthly income to control for residual confounding, along with urbanisation, CCI score, and frequency of outpatient visits. To further understand the impact of severe disorders, all regression analyses were stratified to compare individuals with severe PMHD against the non-PMHD population.

All statistical analyses were performed using SAS software, version 9.4 (SAS Institute Inc., Cary, NC, USA), with statistical significance defined as p-value < 0.05. The study was approved by the Research Ethics Committee of China Medical University Hospital, Taiwan (No. CMUH114-REC2–023), and the requirement for informed consent was waived as this study solely used deidentified secondary data. Data were accessed for research purposes on 10 February 2025. The authors did not have access to information that could identify individual participants during or after data collection.

## Results

### Cancer stage at diagnosis

Between 2016 and 2021, a total of 77,417 women were newly diagnosed with breast cancer. Of these, 12,480 (16.1%) had PMHD, including 1,635 who were classified as having severe disorders. Women with PMHD were significantly older than those without. Notably, those with severe PMHD represented a particularly vulnerable subgroup with significantly higher proportions of low income and severe comorbidities (S3 Table). Despite these adverse characteristics and health conditions, after adjusting for personal and clinical covariates, women with PMHD were significantly less likely to be diagnosed at metastatic breast cancer (Stage IV: 7.3% vs 8.7%; aOR = 0.72, 95% CI = 0.67–0.78). Crucially, this inverse association with metastatic breast cancer diagnosis persisted even among those with severe PMHD (aOR = 0.79, 95% CI = 0.66–0.94) (Table 1).

**Table 1:** Association between PMHD and stage at breast cancer diagnosis.

Variables	Stage at breast cancer diagnosis					Regression 1						Regression 2					
						Without vs with PMHD						Without vs with severe PMHD					
	Stage I, II & III		Stage IV		p value	(ROC = 0.597)						(N = 66,572; ROC = 0.599)					
	n	%	n	%		OR	95% CI			p-value	VIF	OR	95% CI			p-value	VIF
Total	70,880	91.6	6,537	8.4													
PMHD					<0.001						1.039						1.015
Without (ref.)	59,307	91.3	5,630	8.7		1.00						1.00					
With PMHD	11,573	92.7	907	7.3		0.72	0.67	–	0.78	<0.001		–		–		–	
With severe PMHD	1,498	–	137	–		–		–		–		0.79	0.66	–	0.94	0.009	
Age at diagnosis (years old)					<0.001						1.194						1.176
<45 (ref.)	12,878	93.9	837	6.1		1.00						1.00					
45-54	20,528	93.0	1,543	7.0		1.16	1.07	–	1.27	<0.001		1.19	1.08	–	1.30	<0.001	
55-64	19,800	90.7	2,027	9.3		1.55	1.42	–	1.69	<0.001		1.59	1.45	–	1.74	<0.001	
65+	17,674	89.2	2,130	10.8		1.72	1.57	–	1.88	<0.001		1.77	1.61	–	1.94	<0.001	
Mean ± SD	56.6 ± 12.5		60.0 ± 13.0														
Monthly income (TWD)					<0.001						1.056						1.058
Under 22,000 (ref.)	15,593	88.9	1,945	11.1		1.00						1.00					
22,001-24,000	19,975	91.2	1,932	8.8		0.78	0.72	–	0.83	<0.001		0.77	0.72	–	0.83	<0.001	
24,001-40,100	15,268	92.2	1,300	7.9		0.75	0.69	–	0.80	<0.001		0.73	0.68	–	0.79	<0.001	
Above 40,101	20,044	93.7	1,360	6.4		0.59	0.55	–	0.64	<0.001		0.58	0.54	–	0.63	<0.001	
Urbanisation					<0.001						1.022						1.023
Level 1 (ref.)	22,822	92.4	1,877	7.6		1.00						1.00					
Level 2	24,559	91.6	2,258	8.4		1.10	1.03	–	1.17	0.006		1.09	1.02	–	1.17	0.014	
Level 3	11,345	91.3	1,084	8.7		1.13	1.04	–	1.22	0.003		1.11	1.02	–	1.20	0.020	
Level 4	7,794	90.7	804	9.4		1.19	1.09	–	1.30	<0.001		1.19	1.08	–	1.30	<0.001	
Level 5	803	89.6	93	10.4		1.27	1.01	–	1.58	0.037		1.30	1.03	–	1.64	0.031	
Level 6	1,715	88.4	225	11.6		1.44	1.24	–	1.67	<0.001		1.40	1.19	–	1.65	<0.001	
Level 7	1,842	90.4	196	9.6		1.19	1.02	–	1.39	0.032		1.13	0.95	–	1.34	0.166	
Charlson Comorbidity Score					<0.001						1.157						1.143
0 (ref.)	40,271	92.2	3,397	7.8		1.00						1.00					
1	18,110	91.5	1,677	8.5		1.02	0.96	–	1.08	0.597		1.04	0.97	–	1.11	0.281	
Above 2	12,499	89.5	1,463	10.5		1.17	1.10	–	1.26	<0.001		1.17	1.09	–	1.26	<0.001	
Cancer history					0.003						1.019						1.019
No (ref.)	66,880	91.5	6,226	8.5		1.00						1.00					
Yes	4,000	92.8	311	7.2		0.72	0.64	–	0.82	<0.001		0.72	0.63	–	0.82	<0.001	

ROC: Receiver operating characteristic; OR: Odds ratio; CI: Confidence interval; PMHD: Pre-existing mental health disorder; TWD: New Taiwan Dollar; VIF: Variance inflation factor; SD: Standard deviation.

In parallel, regarding bowel cancer, 13,583 (17.2%) of the 79,142 newly diagnosed individuals had PMHD, with 2,639 classified as having severe disorders. Compared to those without disorders, individuals with PMHD were significantly older and female. Similar to the breast cancer stage cohort, individuals with severe PMHD represented the most vulnerable subgroup, being significantly older and having a higher comorbidity score (S4 Table). However, after controlling for personal and clinical covariates, individuals with general PMHD were significantly less likely to receive a diagnosis of metastatic bowel cancer (Stage IV: 24.4% vs 25.7%; aOR = 0.93, 95% CI = 0.89–0.98). In contrast to the breast cancer cohort, this inverse association was not observed among those with severe PMHD (aOR = 1.04, 95% CI = 0.95–1.14) (Table 2).

**Table 2:** Association between PMHD and stage at bowel cancer diagnosis.

Variables	Stage at bowel cancer diagnosis					Regression 1						Regression 2					
						Without vs with PMHD						Without vs with severe PMHD					
	Stage I, II & III		Stage IV		p value	(ROC = 0.532)						(N = 68,198; ROC = 0.532)					
	n	%	n	%		OR	95% CI			p-value	VIF	OR	95% CI			p-value	VIF
Total	59,011	74.6	20,131	25.4													
PMHD					0.003						1.035						1.020
Without (ref.)	48,746	74.4	16,813	25.7		1.00						1					
With PMHD	10,265	75.6	3,318	24.4		0.93	0.89	–	0.98	0.002		–		–		–	
With severe PMHD	1,935	–	704	–		–		–		–		1.04	0.95	–	1.14	0.402	
Sex					0.546						1.010						1.006
Female	25,520	74.5	8,755	25.5		1.00						1					
Male	33,491	74.7	11,376	25.4		0.98	0.95	–	1.02	0.304		1.00	0.96	–	1.03	0.782	
Age at diagnosis (years old)					0.005						1.143						1.139
<45 (ref.)	3,632	72.8	1,357	27.2		1.00						1					
45-54	8,174	74.0	2,879	26.1		0.94	0.87	–	1.02	0.126		0.94	0.87	–	1.02	0.128	
55-64	14,615	75.0	4,873	25.0		0.89	0.83	–	0.95	<0.001		0.89	0.83	–	0.96	0.002	
65+	32,590	74.7	11,022	25.3		0.89	0.83	–	0.95	<0.001		0.87	0.81	–	0.94	<0.001	
Mean ± SD	66.7 ± 13.5		66.8 ± 14.0														
Monthly income (TWD)					<0.001						1.055						1.058
Under 22,000 (ref.)	15,664	72.3	5,988	27.7		1.00						1					
22,001-24,000	20,064	74.8	6,753	25.2		0.87	0.84	–	0.91	<0.001		0.88	0.84	–	0.92	<0.001	
24,001-40,100	9,752	75.0	3,244	25.0		0.86	0.82	–	0.90	<0.001		0.85	0.81	–	0.90	<0.001	
Above 40,101	13,531	76.6	4,146	23.5		0.79	0.76	–	0.83	<0.001		0.79	0.75	–	0.83	<0.001	
Urbanisation					0.009						1.024						1.026
Level 1 (ref.)	16,196	75.5	5,245	24.5		1.00						1					
Level 2	18,550	74.1	6,500	26.0		1.07	1.03	–	1.12	<0.001		1.07	1.02	–	1.12	0.003	
Level 3	10,239	74.1	3,578	25.9		1.07	1.02	–	1.13	0.006		1.07	1.02	–	1.13	0.010	
Level 4	8,115	74.8	2,741	25.3		1.04	0.98	–	1.10	0.168		1.05	0.99	–	1.11	0.148	
Level 5	1,292	74.1	451	25.9		1.08	0.96	–	1.21	0.187		1.08	0.95	–	1.22	0.230	
Level 6	2,280	74.4	785	25.6		1.06	0.97	–	1.16	0.212		1.05	0.95	–	1.15	0.348	
Level 7	2,339	73.8	831	26.2		1.09	0.99	–	1.19	0.051		1.07	0.98	–	1.18	0.155	
Charlson Comorbidity Score					0.137						1.120						1.115
0 (ref.)	19,155	74.8	6,447	25.2		1.00						1					
1	16,894	74.1	5,910	25.9		1.05	1.01	–	1.09	0.027		1.06	1.01	–	1.10	0.014	
Above 2	22,962	74.7	7,774	25.3		1.03	0.99	–	1.07	0.212		1.02	0.98	–	1.07	0.314	
Cancer history					<0.001						1.010						1.011
No (ref.)	54,067	74.2	18,756	25.8		1.00						1					
Yes	4,944	78.2	1,375	21.8		0.81	0.76	–	0.86	<0.001		0.80	0.75	–	0.86	<0.001	

ROC: Receiver operating characteristic; OR: Odds ratio; CI: Confidence interval; PMHD: Pre-existing mental health disorder; TWD: New Taiwan Dollar; VIF: Variance inflation factor; SD: Standard deviation.

The sensitivity analysis using the Stages I–II versus Stages III–IV classification showed findings that were generally consistent with those of the primary analysis. For breast cancer, patients with PMHD had significantly lower odds of being diagnosed with Stages III–IV disease (aOR = 0.80; 95% CI = 0.76–0.83). Among women with severe PMHD, the adjusted odds ratio also indicated lower odds of Stages III–IV disease, although the association was not statistically significant (aOR = 0.98; 95% CI = 0.88–1.11). For bowel cancer, the results of the sensitivity analysis were consistent with those of the primary analysis (S5 Table).

### Cancer screening attendance

In 2019, a total of 3,030,228 women in Taiwan were eligible for breast cancer screening. Data from 1,534,698 women were selected for further analysis after matching. Within this cohort, 255,783 women were identified as having PMHD, with 22,036 classified as having severe disorders. Women with severe PMHD represented a socio-economically disadvantaged and higher comorbidity score group. Notably, a significant disparity in healthcare use was observed, with 61.8% of women with severe PMHD falling into the highest quartile of outpatient visits, compared to only 19.7% of those without disorders (S6 Table).

Over a three-year period, 39.6% (n = 608,249) of eligible women engaged with breast cancer screening, and screening attendees exhibited a higher average number of outpatient visits than non-attendees (16.6 vs 10.3 visits/year) (Table 3). After adjusting for personal and clinical covariates, women with overall PMHD had a statistically significantly higher likelihood of participating in breast cancer screening (aOR = 1.04, 95% CI = 1.03–1.05). However, the findings reversed with disease severity. Even after accounting for the possible positive influence of frequent medical visits, women with severe PMHD had significantly lower odds of attending screening compared to the non-PMHD group (aOR = 0.81, 95% CI = 0.79–0.84) (Table 4).

**Table 3:** Average outpatient visits.

Variables	Without PMHD			With PMHD			With Severe PMHD		
	N	Mean	SD	N	Mean	SD	N	Mean	SD
Breast cancer screening									
Not attendance	797,911	10.3	12.1	128,538	24.3	17.5	12,430	28.0	21.0
Attendance	481,004	16.6	13.1	127,245	29.0	17.6	9,606	33.9	20.7
Bowel Cancer screening									
Not attendance	1,461,736	10.5	12.8	229,204	24.6	18.4	24,155	27.4	21.6
Attendance	773,894	18.8	14.6	217,922	30.7	19.2	18,164	35.4	22.2

PMHD: Pre-existing mental health disorder; SD: Standard deviation.

**Table 4:** Association between PMHD and breast cancer screening attendance.

Variables	Breast cancer screening					Regression 1						Regression 2					
						Without vs with PMHD						Without vs with severe PMHD					
	Not attendance		Attendance		p–value	(ROC=0.678)						(ROC=0.682)					
	n	%	n	%		OR	95% CI			p–value	VIF	OR	95% CI			p–value	VIF
Total	9,26,449	60.4	6,08,249	39.6													
PMHD					<0.001						1.123						1.167
Without (ref.)	7,97,911	86.1	4,81,004	79.1		1.00						1.00					
With PMHD	1,28,538	13.9	1,27,245	20.9		1.04	1.03	–	1.05	<0.001		–		–		–	
With severe PMHD	12,430	–	9,606	–								0.81	0.79	–	0.84	<0.001	
Age (years)					<0.001						1.062						1.058
45–49 (ref.)	1,59,578	17.2	1,04,974	17.3		1.00						1.00					
50–54	1,82,925	19.7	1,23,189	20.3		0.94	0.93	–	0.95	<0.001		0.95	0.91	–	0.98	0.005	
55–59	2,03,161	21.9	1,36,367	22.4		0.85	0.84	–	0.86	<0.001		0.85	0.82	–	0.89	<0.001	
60–64	1,90,677	20.6	1,28,877	21.2		0.82	0.81	–	0.83	<0.001		0.82	0.79	–	0.85	<0.001	
64–69	1,90,108	20.5	1,14,842	18.9		0.68	0.67	–	0.69	<0.001		0.68	0.66	–	0.71	<0.001	
Mean ± SD	57.3 ± 7.0		57.2 ± 6.9			–											
Monthly income (TWD)					<0.001						1.036						1.033
Under 23,100	2,97,777	32.1	1,21,029	19.9		1.00						1.00					
23,101–24,000	2,50,732	27.1	1,83,242	30.1		1.4	1.39	–	1.42	<0.001		1.42	1.37	–	1.46	<0.001	
24,001–40,100	1,93,818	20.9	1,46,652	24.1		1.49	1.48	–	1.51	<0.001		1.55	1.49	–	1.60	<0.001	
Above 40,100	1,84,122	19.9	1,57,326	25.9		1.73	1.71	–	1.75	<0.001		1.7	1.64	–	1.76	<0.001	
Urbanisation					<0.001						1.025						1.023
Level 1 (ref.)	2,73,884	29.6	1,88,001	30.9		1.00						1					
Level 2	3,34,417	36.1	2,04,973	33.7		0.91	0.91	–	0.92	<0.001		0.91	0.88	–	0.94	<0.001	
Level 3	1,47,520	15.9	96,210	15.8		0.93	0.92	–	0.94	<0.001		0.91	0.88	–	0.94	<0.001	
Level 4	1,06,421	11.5	72,863	12.0		0.95	0.94	–	0.96	<0.001		0.91	0.88	–	0.95	<0.001	
Level 5	11,890	1.3	8,744	1.4		1.00	0.97	–	1.03	0.791		0.97	0.88	–	1.07	0.498	
Level 6	25,191	2.7	18,026	3.0		0.99	0.97	–	1.01	0.408		0.98	0.92	–	1.05	0.601	
Level 7	27,126	2.9	19,432	3.2		0.99	0.97	–	1.01	0.253		0.95	0.89	–	1.02	0.173	
CCI					<0.001						1.371						1.443
0 (ref.)	6,34,238	68.5	3,23,573	53.2		1.00						1.00					
1	1,68,728	18.2	1,60,568	26.4		1.16	1.15	–	1.17	<0.001		1.19	1.16	–	1.23	<0.001	
Above 2	1,23,483	13.3	1,24,108	20.4		1.10	1.08	–	1.11	<0.001		1.06	1.03	–	1.1	0.001	
Average annual outpatient visits					<0.001						1.479						1.566
<Q1 (Low)	3,35,552	36.2	61,285	10.1		1.00						1.00					
Q1~Q3 (Medium)	4,11,479	44.4	3,34,921	55.1		4.05	4.01	–	4.09	<0.001		4.11	3.97	–	4.26	<0.001	
>Q3 (High)	1,79,418	19.4	2,12,043	34.9		6.01	5.93	–	6.08	<0.001		6.24	5.97	–	6.51	<0.001	

1. Q1 = 4, Q2 = 11, Q3 = 21

2. ROC: Receiver operating characteristic; OR: Odds ratio; CI: Confidence interval; PMHD: Pre-existing mental health disorder; TWD: New Taiwan Dollar; SD: Standard deviation.

In parallel, in 2019, a total of 5,453,628 individuals in Taiwan were eligible for bowel cancer screening, of whom 2,682,756 were included post-matching in the analyses. Among this subgroup, 447,126 individuals had a documented history of mental health disorders, with 42,319 classified as having severe PMHD. Similar to the breast cancer screening cohort, individuals with severe PMHD had higher proportions of lower monthly income and higher CCI scores (S7 Table). A distinct pattern was also observed, with 60.8% of individuals with severe PMHD falling into the highest quartile of outpatient visits, representing a rate nearly three times that of the non-PMHD group (22.2%).

Overall, 37% of study participants underwent FIT within the three-year study period. Similar to findings in breast cancer screening, attendees generally had a higher average number of outpatient visits than non-attendees (Table 3). Frequent outpatient visits exhibited a strong positive association with screening participation (aOR = 6.57 for >Q3 vs < Q1) (Table 5). Consistent with this pattern, individuals with overall PMHD, who generally used healthcare services more frequently, had a significantly higher likelihood of participating in bowel cancer screening compared to those with no disorders (aOR = 1.12, 95% CI = 1.11–1.12). However, this association was not observed in those with severe disorders. Individuals with severe PMHD were significantly less likely to undergo FIT screening (aOR = 0.88, 95% CI = 0.86–0.90) (Table 5).

**Table 5:** Association between PMHD and bowel cancer screening attendance.

Variables	Bowel cancer screening					Regression 1						Regression 2					
						Without vs with PMHD						Without vs with severe PMHD					
	Not attendance		Attendance		p-value	(ROC = 0.705)						(ROC = 0.714)					
	n	%	n	%		OR	95% CI			p-value	VIF	OR	95% CI			p-value	VIF
Total	1,690,940	63.0	991,816	37.0													
PMHD					<0.001						1.112						1.150
Without (ref.)	1,461,736	86.5	773,894	78.0		1.00						1.00					
With PMHD	229,204	13.6	217,922	22.0		1.12	1.11	–	1.12	<0.001		–		–		–	
With severe PMHD	24,155	–	18,164	–								0.88	0.86	–	0.90	<0.001	
Sex					<0.001						1.022						1.030
Male (ref.)	823,635	48.7	389,913	39.3		1.00											
Female	867,305	51.3	601,903	60.7		1.36	1.35	–	1.37	<0.001		1.36	1.34	–	1.39	<0.001	
Age (years)					<0.001						1.078						1.065
50-54 (ref.)	402,228	23.8	192,810	19.4		1.00											
55-59	404,329	23.9	227,297	22.9		1.07	1.06	–	1.08	<0.001		1.09	1.06	–	1.12	<0.001	
60-64	348,322	20.6	231,494	23.3		1.18	1.17	–	1.19	<0.001		1.20	1.17	–	1.23	<0.001	
64-69	309,033	18.3	228,447	23.0		1.21	1.20	–	1.22	<0.001		1.18	1.15	–	1.22	<0.001	
70-74	227,028	13.4	111,768	11.3		0.72	0.71	–	0.73	<0.001		0.72	0.69	–	0.74	<0.001	
Mean ± SD	60.6 ± 6.9		61.1 ± 6.5														
Monthly income (TWD)					<0.001						1.060						1.048
Under 23,100	559,562	33.1	209,566	21.1		1.00											
23,101−24,000	434,457	25.7	320,763	32.3		1.42	1.41	–	1.43	<0.001		1.42	1.39	–	1.45	<0.001	
24,001-40,100	316,796	18.7	215,164	21.7		1.44	1.43	–	1.45	<0.001		1.43	1.39	–	1.46	<0.001	
Above 40,100	380,125	22.5	246,323	24.8		1.39	1.38	–	1.40	<0.001		1.43	1.39	–	1.46	<0.001	
Urbanisation					<0.001						1.027						1.024
Level 1 (ref.)	487,444	28.8	275,026	27.7		1.00											
Level 2	585,778	34.6	329,048	33.2		1.03	1.02	–	1.04	<0.001		1.02	0.99	–	1.04	0.183	
Level 3	276,620	16.4	164,867	16.6		1.04	1.03	–	1.05	<0.001		1.03	1.00	–	1.06	0.025	
Level 4	205,105	12.1	131,116	13.2		1.09	1.08	–	1.10	<0.001		1.08	1.05	–	1.11	<0.001	
Level 5	26,029	1.5	17,994	1.8		1.13	1.11	–	1.16	<0.001		1.06	0.99	–	1.14	0.076	
Level 6	54,605	3.2	37,220	3.8		1.15	1.13	–	1.17	<0.001		1.11	1.06	–	1.17	<0.001	
Level 7	55,359	3.3	36,545	3.7		1.09	1.08	–	1.11	<0.001		1.07	1.02	–	1.12	0.005	
Charlson Comorbidity Score					<0.001						1.385						1.470
0 (ref.)	1,193,506	70.6	509,578	51.4		1.00											
1	285,201	16.9	262,354	26.5		1.22	1.21	–	1.23	<0.001		1.17	1.15	–	1.20	<0.001	
Above 2	212,233	12.6	219,884	22.2		1.23	1.22	–	1.24	<0.001		1.16	1.13	–	1.19	<0.001	
Average annual outpatient visits					<0.001						1.424						1.599
<Q1 (Low)	645,534	38.2	91,402	9.2		1.00											
Q1 ~ Q3 (Medium)	703,685	41.6	496,747	50.1		4.19	4.16	–	4.23	<0.001		4.56	4.44	–	4.69	<0.001	
>Q3 (High)	341,721	20.2	403,667	40.7		6.57	6.51	–	6.64	<0.001		7.51	7.27	–	7.75	<0.001	

1. Q1 = 4, Q2 = 12, Q3 = 23

2. ROC: Receiver operating characteristic; OR: Odds ratio; CI: Confidence interval; PMHD: Pre-existing mental health disorder; TWD: New Taiwan Dollar; SD: Standard deviation.

## Discussion

This population-based study reveals distinct cancer outcomes under Taiwan’s highly accessible universal healthcare system. Unlike disparities reported elsewhere, overall PMHD was associated with higher screening participation and reduced odds of metastatic stage diagnosis for both breast and bowel cancers. However, these advantages were largely absent for individuals with severe PMHD, despite their more frequent healthcare use. These findings suggest that while high healthcare accessibility may contribute to a potential surveillance effect, it may remain insufficient to mitigate the profound barriers associated with severe mental illness.

### Potential surveillance effect

Our findings regarding higher screening uptake and reduced odds of metastatic disease at diagnosis among individuals with overall PMHD differ from previous literature. Reviews from other countries have synthesised findings to show that mental illnesses are associated with reduced screening participation and advanced stage at diagnosis for breast or bowel cancers [2,9,18]. Other studies attributed these disparities to cognitive disturbance [16,17] social isolation [14,16,17], transportation difficulties and costs [5,13,14,17], quality of the doctor–patient relationship [12,14,17], and fragmented healthcare services [6,14,15,17,18]. In contrast, Taiwan has no strict distinction between primary care and specialist care, and GP referrals to secondary care are not mandatory. This accessibility contributes to an average of 13.12 outpatient visits per person [23], double the Organisation for Economic Co-operation and Development average of 6.5 [29], thereby increasing exposure to healthcare services. We hypothesise that this distinct high frequency of contact may contribute to a potential surveillance effect for individuals with PMHD (Fig 1).

**Fig 1:**
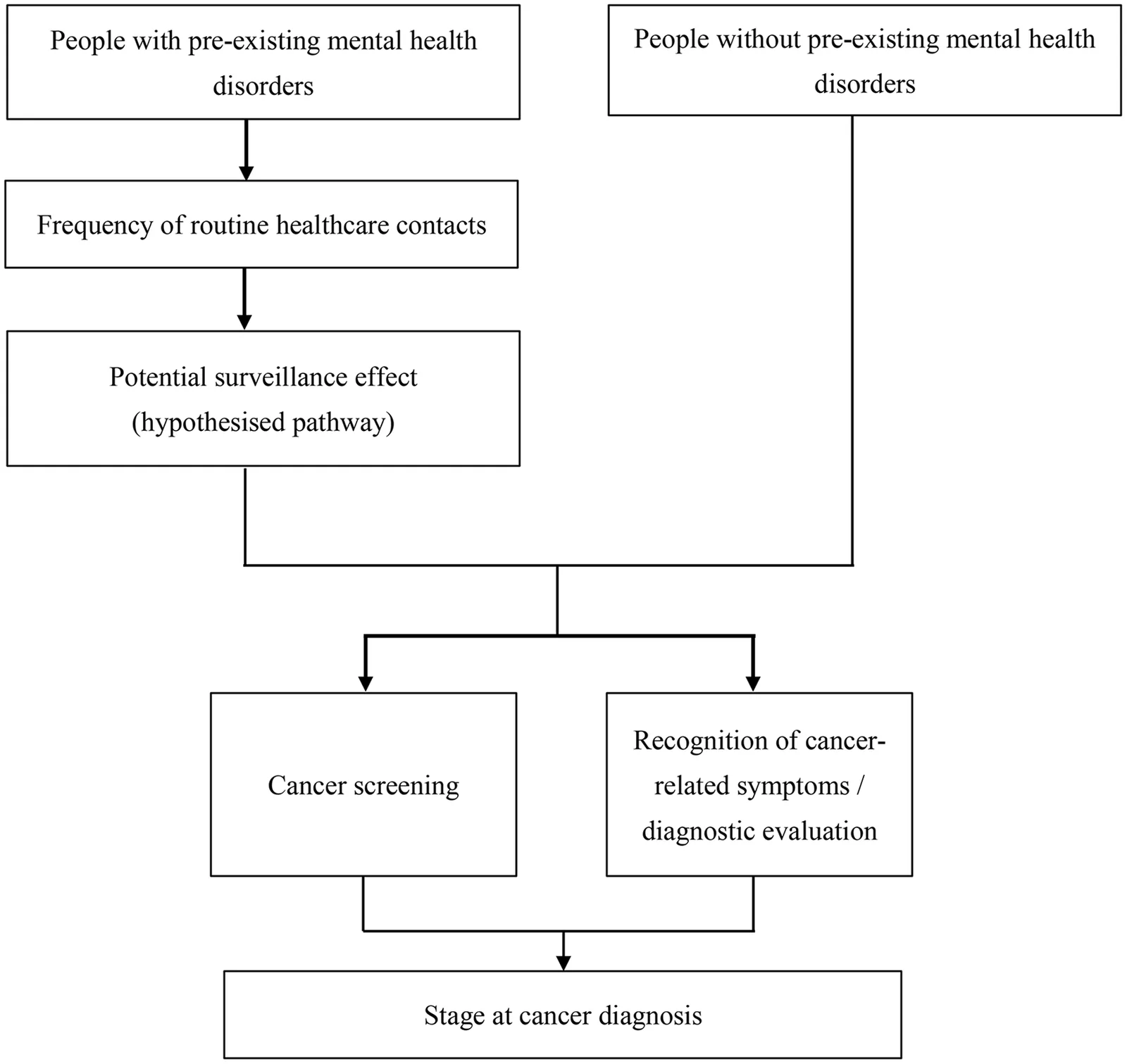
Conceptual framework illustrating the hypothesised surveillance effect associated with differences in routine healthcare contact frequency between individuals with and without pre-existing mental health disorders.

Within this accessible healthcare system, the specific advantage for individuals with PMHD is likely due to increased exposure to screening information through their frequent clinical contact. Our data indicate that individuals with PMHD attended outpatient visits significantly more frequently than those without PMHD. Screening attendees averaged 29–31 visits per year, compared with 17–19 visits among the non-PMHD group (Table 3). This high frequency may facilitate screening participation through two complementary mechanisms. Firstly, patients may encounter health promotion materials within the clinical environment, such as posters detailing breast and bowel cancer screening displayed in clinics and hospitals. Evidence shows that screening participation and knowledge consistently increased with the frequency of exposure to screening information [30]. Secondly, patients are subject to active systemic prompts, rather than relying solely on reminders from physicians during visits. Screening reminders are also automatically printed on prescriptions at each visit, which has been shown to effectively increase screening participation [31]. Consequently, frequent healthcare contact may increase opportunities to receive screening information through health education or recommendations from healthcare professionals. Notably, this systemic advantage may extend from screening participation to the diagnostic stage. The high frequency of healthcare visits may also increase the likelihood of timely recognition of alarm symptoms, such as palpable lumps or rectal bleeding, and subsequent referrals for examinations. These two potential mechanisms, including enhanced screening uptake and increased opportunities for symptom recognition, provide a possible hypothesis for the reduced odds of metastatic disease at diagnosis observed in the overall PMHD group.

### Challenges for individuals with severe illnesses

However, contrasting findings for screening participation were found among people with a history of hospital admissions or emergency department visits for mental health disorders. Screening attendees with severe PMHD averaged over 33 visits per year, representing the highest among all groups (Table 3). Despite high medical use, these individuals were significantly less likely to attend breast or bowel cancer screening, which aligns with prior studies [14,31]. We therefore hypothesise that the advantage of frequent healthcare visits may not suffice to mitigate the barriers experienced by people with a diagnosis of severe mental disorders.

Individuals with severe disorders often have insufficient support from friends, family, or healthcare professionals, despite the demonstrated benefits of such support [6,15]. This may be particularly problematic for those who do not know what to expect during screening [15]. Needs for assistance in arranging cancer screening appointments have been revealed among this group [5,14,31]. In addition, individuals with a diagnosis of severe mental health disorders, their treating physicians, and other health practitioners, often focus on immediate medical concerns and complex mental health needs over the possibility of taking part in screening [6,12,13,15,18]. As a result, physicians may prioritise the management of acute mental health symptoms, leading to long-term preventive measures being overlooked or deprioritised during consultations [6,18]. For example, people with mental health disorders and red-flag symptoms are significantly less likely to receive prompt endoscopic investigations and experience diagnostic intervals that are more than twice as long as the general population [4].

Furthermore, symptoms of mental disorders, including depression, anxiety, and cognitive impairment, can hinder navigation of healthcare pathways and reduce motivation to attend preventive appointments [10,14,15,18]. This lack of engagement is particularly evident in bowel cancer screening. FIT-based screening requires home-based sample collection and return, which may evoke negative emotions such as disgust or shame and create difficulties in following sampling guidelines, thereby reducing participation [11,16,32,33]. Similarly, while breast cancer screening is professionally administered, individuals with mental health disorders have reported that participation is often inhibited by a lack of trust in unfamiliar staff, the anticipation of a traumatising procedure, and the fear of a cancer diagnosis [6,14,15].

### Strengths and limitations

This study has several strengths. Firstly, the nationwide population-based design covers 99% of the Taiwanese population, minimising selection bias. Secondly, to our knowledge, this is the first study in Asia to simultaneously examine disparities at two critical phases of the cancer care pathway, including screening attendance and stage at diagnosis, within a universal healthcare system. This design allows for a more comprehensive understanding of potential inequality in cancer care among people with PMHD. Finally, the inclusion of both breast and bowel cancers enables a novel comparison between distinct screening modalities. While mammography is a professionally administered procedure performed entirely within clinical facilities, bowel cancer screening using FIT requires self-administration, involving home-based sample collection and return. By presenting these outcomes, our study provides insights into the association between mental illness and the differing engagement mechanisms screening requires.

However, several limitations should be noted. NHIRD relies on administrative claims data rather than medical charts. Therefore, we could not access physicians’ notes to verify the severity of mental symptoms or the exact reasons for non-attendance at screening. In addition, information on several potentially important factors was unavailable, including medication adherence, caregiver or family support, lifestyle factors such as smoking and alcohol consumption, sociodemographic factors such as educational attainment and marital status, psychiatric disease duration, and functional status. These factors may influence cancer screening behaviour and cancer stage at diagnosis and could therefore result in residual confounding. Moreover, NHIRD does not capture out-of-pocket healthcare services, potentially leading to an underestimation of overall screening participation. This missing information likely more often affects the non-PMHD group, as they may have greater access to unrecorded private screening. Consequently, our findings primarily reflect participation in government-funded screenings, suggesting a potentially greater dependence on government-subsidised screening services for the PMHD group.

Our definition of PMHD required a psychiatric diagnosis accompanied by psychotropic medication use and was intended to identify individuals with clinically recognised psychiatric conditions receiving pharmacological treatment using routinely available claims data. Nevertheless, this claims-based definition may not fully capture the spectrum of PMHD, particularly among individuals with psychiatric conditions who were not receiving pharmacological treatment. In addition, combining different psychiatric disorders into a single PMHD category may mask clinically meaningful differences in healthcare-seeking behaviours and barriers to cancer care. Further research is needed to explore whether cancer care disparities vary across specific mental health conditions and across different sex and ethnic groups which was not examined in this analysis.

The definition of severe PMHD relied on psychiatric hospitalisation or emergency department visits, which may serve as proxies for illness severity but do not directly measure psychiatric symptom severity or functional impairment. Therefore, some individuals with severe mental illnesses managed exclusively in outpatient settings may have been classified as having non-severe PMHD.

Excluding cases with missing stages or CIS could theoretically introduce selection bias. However, a supplementary analysis (S8 Table) comparing the excluded cohorts indicates minimal selection bias, suggesting that the observed surveillance effect may even be underestimated. Moreover, the relatively low discriminatory performance of the regression models, with ROC values of approximately 0.53–0.60, indicates that important determinants of cancer stage may not have been captured in the available datasets. In addition, although the associations between PMHD and advanced-stage cancer diagnosis were statistically significant, the magnitude of the observed associations was modest, particularly for bowel cancer (aOR = 0.93). Therefore, the clinical relevance of these findings should be interpreted with caution.

Finally, the three-year observation period (2019–2021) was selected to allow sufficient time to capture delayed or catch-up screening during the COVID-19 pandemic. However, this extended observation period may result in a higher cumulative proportion of screening participation than would be observed within a strict two-year interval and could therefore overestimate adherence to the recommended biennial screening schedule.

### Clinical and research implications

Our findings suggest that an accessible universal healthcare system may mitigate health disparities for people with PMHD. The outcomes observed in cancer screening and staging for the overall PMHD population suggest that greater accessibility may help reduce disparities. For policymakers in countries with strict gatekeeping, Taiwan’s experience may provide support for policies that facilitate barrier-free access to medical professionals. However, universal healthcare coverage alone may not mitigate disparities among individuals with severe disorders. Our results demonstrate that frequent healthcare visits did not translate into better outcomes for those with severe mental disorders, highlighting the limitations of relying solely on a high-access system without more specific and targeted support.

Despite averaging over 33 outpatient visits annually, individuals with severe disorders remained significantly less likely to undergo screening. This highlights potential need for active interventions and procedural simplification. As potential future strategies, cancer patient navigators or specialist screening practitioners could provide cancer screening education and coordinate appointments, particularly for patients with functional impairments or insufficient social support. Moreover, people with lived experienced of mental health disorders (such as peer support workers or peer advocates) might also provide psychosocial support to facilitate engagement with screening. To address the logistical barriers of bowel cancer screening using FIT, future interventions could also evaluate the feasibility of “on-site” screening strategies. Collaborating with community care networks to facilitate local or postal sample return or providing facilities and assistance for patients to complete stool collections directly at the hospital during visits may reduce the challenges of bowel cancer screening. However, the effectiveness and acceptability of these approaches require evaluation in future research.

Moreover, the disparity in metastatic bowel cancer diagnosis and a higher proportion of stage unknown (S8 Table) for the severe disorders group highlights the necessity for targeted support to help these individuals. Further research is needed to explore the experiences and perspectives of people with severe PMHD, particularly their perceived barriers and enablers to physical health screening. Understanding these experiences may help inform the development of targeted interventions that are responsive to the needs of this population.

## Conclusion

This nationwide study found that, within Taiwan’s universal healthcare system, people with PMHD generally had higher screening participation and lower odds of metastatic disease at diagnosis for breast and bowel cancers. However, those with severe PMHD had lower screening participation and remained at higher risk of metastatic bowel cancer at diagnosis. The universal healthcare system alone may be insufficient to mitigate disparities in cancer screening and stage at diagnosis among people with severe PMHD. Targeted interventions should therefore complement universal healthcare access, including simplified screening procedures and multidisciplinary, person-centred care, to promote greater equity in cancer screening participation and reduce disparities in stage at diagnosis.

## Supporting information

S1 Table Included mental health disorders. (DOCX)

S2 Table Balance diagnostics before and after matching. (DOCX)

S3 Table Characteristics among women newly diagnosed with breast cancer. (DOCX)

S4 Table Characteristics among people newly diagnosed with bowel cancer. (DOCX)

S5 Table Sensitivity analysis for cancer stage at diagnosis. (DOCX)

S6 Table Characteristics among women eligible for breast cancer screening. (DOCX)

S7 Table Characteristics among people eligible for bowel cancer screening. (DOCX)

S8 Table Comparison of reasons for exclusion (carcinoma in situ versus missing stage) between individuals with and without PMHD. (DOCX)

S1 Fig Framework for stage at cancer diagnosis analysis. (DOCX)

S2 Fig Flowchart of study subjects for breast cancer diagnosis analysis. (DOCX)

S3 Fig Flowchart of study subjects for bowel cancer diagnosis analysis. (DOCX)

S4 Fig Framework for cancer screening attendance analysis. (DOCX)

S5 Fig Flowchart of study subjects for breast cancer screening analysis. (DOCX)

S6 Fig Flowchart of study subjects for bowel cancer screening analysis. (DOCX)
