# Supplementary material for: Disparities in screening participation and stage at diagnosis for breast and bowel cancers among individuals with pre-existing mental health disorders: A population-based study
Source: PLoS One. 2026 Oct 27;21(10):e0361617. doi: 10.1371/journal.pone.0361617 (PMC13712726; doi:10.1371/journal.pone.0361617)
Supplement: Supplementary file 1 [file pone.0361617.s001.docx]

| **Category** | **ICD 9** | **ICD 10** |
| --- | --- | --- |
| Anxiety Disorders | 293.84, 300, 300.01, 300.02, 300.09, 300.22, 300.23, 300.29, 309.21, 313.23 | F06.4, F40.00, F40.10, F40.218, F40.228, F40.23x, F40.248, F40.298, F41.0, F41.1 F41.8, F41.9, F93.0, F94.0 |
| Bipolar and Related Disorders | 293.83, 296.40, 296.41, 296.42, 296.43, 296.44, 296.45, 296.46, 296.50, 296.51, 296.52, 296.53, 296.54, 296.55, 296.56, 296.7, 296.8, 296.89, 301.13 | F06.33, F06.34F31.0, F31.11, F31.12, F31.13, F31.2, F31.31, F31.32, F31.4, F31.5, F31.71, F31.72, F31.73, F31.74, F31.75, F31.76, F 31.81, F31.89, F31.9, F34.0, F34.8, F34.81 |
| Depressive Disorders | 293.83, 296.21, 296.22, 296.23, 296.24, 296.25, 296.26, 296.20, 296.31, 296.32, 296.33, 296.34, 296.35, 296.36, 296.30, 296.99, 300.4, 311, 625.4 | F06.31, F06.32, F06.34, F32.0, F32.1, F32.2, F32.3, F32.4, F32.5, F32.8, F32.81, F32.89, F32.9, F33.0, F33.1, F33.2, F33.3, F33.41, F33.42, F33.9, F34.1, F34.8, F34.81, N94.3 |
| Dissociative Disorders | 300.12, 300.14, 300.15, 300.6 | F44.0, F44.81, F44.89, F44.9, F48.1 |
| Feeding and Eating Disorders | 307.1, 307.5, 307.51, 307.52, 307.53, 307.59 | F50.01, F50.02, F50.2, F50.8, F50.81, F50.82, F50.89, F50.9, F98.21, F98.3 |
| Obsessive-Compulsive and Related Disorders | 294.8, 300.3, 300.7, 312.39, 698.4 | F06.8, F42, F42.2, F42.3, F42.4, F42.8, F42.9, F45.22, F63.3, L98.1 |
| Personality Disorders | 301, 301.2, 301.22, 301.4, 301.5, 301.6, 301.7, 301.81, 301.82, 301.83, 301.89, 301.9, 310.1 | F07.0, F21, F60.0, F60.1, F60.2, F60.3, F60.4, F60.5, F60.6, F60.7, F60.81, F60.89, F60.9 |
| Schizophrenia Spectrum and Other Psychotic Disorders | 293.81, 293.82, 293.89, 295.4, 295.70, 295.9, 297.1, 298.8, 298.9, 301.22, 781.99 | F06.0 F06.1, F06.2, F20.81, F20.9, F21, F22, F23, F25.0, F25.1, F28, F29, R29.818 |
| Substance-Related and Addictive Disorders | 291.81, 291.9, 292, 292.0, 292.89, 292.9, 303, 303.90, 304.00, 304.10, 304.20, 304.40, 304.50, 304.60, 304.90, 305.00, 305.30, 305.50, 305.1, 305.40, 305.60, 305.70, 305.90, 305.9, 305.90, 312.31 | F10.10, F10.11, F10.129, F10.20, F10.21, F10.229, F10.232, F10.239, F10.929, F10.99, F11.10, F11.11, F11.122, F11.129, F11.20, F11.21, F11.222, F11.229, F11.23, F11.922, F11.929, F11.93, F11.99, F12.122, F12.129, F12.222, F12.229, F12.23, F12.922, F12.929, F12.93, F12.99, F13.10, F13.11, F13.129, F13.20, F13.21, F13.229, F13.232, F13.239, F13.929, F13.932, F13.939, F13.99, F14.10, F14.11, F14.122, F14.129, F14.20, F14.21, F14.222, F14.229, F14.23, F14.922, F14.929, F14.99, F15.10, F15.11, F15.122, F15.129, F15.20, F15.21, F15.222, F15.229, F15.23, F15.922, F15.929, F15.93, F15.99, F16.10, F16.11, F16.129, F16.20, F16.21, F16.229, F16.929, F16.983, F16.99, F17.200, F17.201, F17.203, F17.209, F18.10, F18.11, F18.129, F18.20, F18.21, F18.229, F18.929, F18.99, F19.10, F19.11, F19.129, F19.20, F19.21, F19.229, F19.239, F19.929, F19.939, F19.99, F63.0, Z72.0 |
| Trauma- and Stressor-Related Disorders | 308.3, 309.0, 309.24, 309.28, 309.3, 309.4, 309.81, 309.89, 309.9, 313.89 | F43.0, F43.10, F43.20, F43.21, F43.22, F43.23, F43.24, F43.25, F43.8, F43.9, F94.1, F94.2 |
