# Supplementary material for: Disparities in screening participation and stage at diagnosis for breast and bowel cancers among individuals with pre-existing mental health disorders: A population-based study
Source: PLoS One. 2026 Oct 27;21(10):e0361617. doi: 10.1371/journal.pone.0361617 (PMC13712726; doi:10.1371/journal.pone.0361617)
Supplement: Supplementary file 2 [file pone.0361617.s002.docx]

| Variables | Before matching | | | | | After matching | | | | |
| --- | --- | --- | --- | --- | --- | --- | --- | --- | --- | --- |
|  | Without PMHD | | With PMHD | | SMD | Without PMHD | | With PMHD | | SMD |
|  | N | % | N | % |  | N | % | N | % |  |
| ***Breast cancer screening cohort*** |  |  |  |  |  |  |  |  |  |  |
| Age (years) |  |  |  |  |  |  |  |  |  |  |
| 45-49 | 645,918 | 23.28 | 44,092 | 17.24 | -0.151 | 220,460 | 17.24 | 44,092 | 17.24 | 0.000 |
| 50-54 | 613,124 | 22.10 | 51,019 | 19.95 | -0.053 | 255,095 | 19.95 | 51,019 | 19.95 | 0.000 |
| 55-59 | 587,816 | 21.19 | 56,588 | 22.12 | 0.023 | 282,940 | 22.12 | 56,588 | 22.12 | 0.000 |
| 60-64 | 517,618 | 18.66 | 53,259 | 20.82 | 0.054 | 266,295 | 20.82 | 53,259 | 20.82 | 0.000 |
| 64-69 | 409,969 | 14.78 | 50,825 | 19.87 | 0.135 | 254,125 | 19.87 | 50,825 | 19.87 | 0.000 |
| Mean ± SD | 55.93 ± 6.98 | | 57.31 ± 6.97 | | 0.198 | 57.25 ± 6.98 | | 57.31 ± 6.97 | | 0.009 |
| Monthly income (TWD) |  |  |  |  |  |  |  |  |  |  |
| Under 23,100 | 835,589 | 30.12 | 69,801 | 27.29 | -0.063 | 349,005 | 27.29 | 69,801 | 27.29 | 0.000 |
| 23,101-24,000 | 646,628 | 23.31 | 72,329 | 28.28 | 0.114 | 361,645 | 28.28 | 72,329 | 28.28 | 0.000 |
| 24,001-40,100 | 617,652 | 22.26 | 56,745 | 22.18 | -0.002 | 283,725 | 22.18 | 56,745 | 22.18 | 0.000 |
| Above 40,100 | 674,576 | 24.31 | 56,908 | 22.25 | -0.049 | 284,540 | 22.25 | 56,908 | 22.25 | 0.000 |
| ***Bowel cancer screening cohort*** |  |  |  |  |  |  |  |  |  |  |
| Sex |  |  |  |  |  |  |  |  |  |  |
| Male | 2,648,161 | 52.89 | 202,258 | 45.24 | -0.154 | 1,011,290 | 45.24 | 202,258 | 45.24 | 0.000 |
| Female | 2,358,341 | 47.11 | 244,868 | 54.76 | 0.154 | 1,224,340 | 54.76 | 244,868 | 54.76 | 0.000 |
| Age (years) |  |  |  |  | 0.154 |  |  |  |  | 0.000 |
| 50-54 | 1,285,925 | 25.69 | 99,173 | 22.18 | -0.082 | 495,865 | 22.18 | 99,173 | 22.18 | 0.000 |
| 55-59 | 1,253,736 | 25.04 | 105,271 | 23.54 | -0.035 | 526,355 | 23.54 | 105,271 | 23.54 | 0.000 |
| 60-64 | 1,105,254 | 22.08 | 96,636 | 21.61 | -0.011 | 483,180 | 21.61 | 96,636 | 21.61 | 0.000 |
| 64-69 | 873,291 | 17.44 | 89,580 | 20.03 | 0.066 | 447,900 | 20.03 | 89,580 | 20.03 | 0.000 |
| 70-74 | 488,296 | 9.75 | 56,466 | 12.63 | 0.091 | 282,330 | 12.63 | 56,466 | 12.63 | 0.000 |
| Mean ± SD | 59.95 ± 6.57 | | 60.82 ± 6.73 | | 0.131 | 60.78 ± 6.73 | | 60.82 ± 6.73 | | 0.006 |
| Monthly income (TWD) |  |  |  |  |  |  |  |  |  |  |
| Under 23,100 | 1,650,756 | 32.97 | 128,188 | 28.67 | -0.093 | 640,940 | 28.67 | 128,188 | 28.67 | 0.000 |
| 23,101-24,000 | 1,129,077 | 22.55 | 125,870 | 28.15 | 0.129 | 629,350 | 28.15 | 125,870 | 28.15 | 0.000 |
| 24,001-40,100 | 963,874 | 19.25 | 88,660 | 19.83 | 0.015 | 443,300 | 19.83 | 88,660 | 19.83 | 0.000 |
| Above 40,100 | 1,262,795 | 25.22 | 104,408 | 23.35 | -0.044 | 522,040 | 23.35 | 104,408 | 23.35 | 0.000 |

Note: PMHD: Pre-existing mental health disorder; TWD: New Taiwan Dollar; SD: Standard deviation; SMD: Standardised mean differences.
