# Supplementary material for: Disparities in screening participation and stage at diagnosis for breast and bowel cancers among individuals with pre-existing mental health disorders: A population-based study
Source: PLoS One. 2026 Oct 27;21(10):e0361617. doi: 10.1371/journal.pone.0361617 (PMC13712726; doi:10.1371/journal.pone.0361617)
Supplement: Supplementary file 3 [file pone.0361617.s003.docx]

| Variables | Total | | Without PMHD | | With PMHD | | With Severe PMHD | |
| --- | --- | --- | --- | --- | --- | --- | --- | --- |
|  | n | % | n | % | n | % | n | % |
| Total | 77,417 | 100.0 | 64,937 | 100.0 | 12,480 | 100.0 | 1,635 | 100.0 |
| Age at diagnosis (years old) |  |  |  |  |  |  |  |  |
| <45 | 13,715 | 17.7 | 12,547 | 19.3 | 1,168 | 9.4 | 152 | 9.3 |
| 45-54 | 22,071 | 28.5 | 19,297 | 29.7 | 2,774 | 22.2 | 364 | 22.3 |
| 55-64 | 21,827 | 28.2 | 18,188 | 28.0 | 3,639 | 29.2 | 444 | 27.2 |
| 65+ | 19,804 | 25.6 | 14,905 | 23.0 | 4,899 | 39.3 | 675 | 41.3 |
| Mean ± SD | 56.9 ± 12.6 | | 56.0 ± 12.3 | | 61.8 ± 12.8 | | 63.0 ± 14.1 | |
| Monthly income (TWD) |  |  |  |  |  |  |  |  |
| Under 22,000 | 17,538 | 22.7 | 13,860 | 21.3 | 3,678 | 29.5 | 657 | 40.2 |
| 22,001-24,000 | 21,907 | 28.3 | 18,154 | 28.0 | 3,753 | 30.1 | 458 | 28.0 |
| 24,001-40,100 | 16,568 | 21.4 | 14,365 | 22.1 | 2,203 | 17.7 | 225 | 13.8 |
| Above 40,101 | 21,404 | 27.7 | 18,558 | 28.6 | 2,846 | 22.8 | 295 | 18.0 |
| Urbanization |  |  |  |  |  |  |  |  |
| Level 1 | 24,699 | 31.9 | 20,803 | 32.0 | 3,896 | 31.2 | 473 | 28.9 |
| Level 2 | 26,817 | 34.6 | 22,478 | 34.6 | 4,339 | 34.8 | 576 | 35.2 |
| Level 3 | 12,429 | 16.1 | 10,588 | 16.3 | 1,841 | 14.8 | 239 | 14.6 |
| Level 4 | 8,598 | 11.1 | 7,111 | 11.0 | 1,487 | 11.9 | 212 | 13.0 |
| Level 5 | 896 | 1.2 | 732 | 1.1 | 164 | 1.3 | 21 | 1.3 |
| Level 6 | 1,940 | 2.5 | 1,567 | 2.4 | 373 | 3.0 | 55 | 3.4 |
| Level 7 | 2,038 | 2.6 | 1,658 | 2.6 | 380 | 3.0 | 59 | 3.6 |
| Charlson Comorbidity Score |  |  |  |  |  |  |  |  |
| 0 | 43,668 | 56.4 | 38,576 | 59.4 | 5,092 | 40.8 | 533 | 32.6 |
| 1 | 19,787 | 25.6 | 16,081 | 24.8 | 3,706 | 29.7 | 452 | 27.7 |
| Above 2 | 13,962 | 18.0 | 10,280 | 15.8 | 3,682 | 29.5 | 650 | 39.8 |
| Cancer history |  |  |  |  |  |  |  |  |
| No | 73,106 | 94.4 | 61,513 | 94.7 | 11,593 | 92.9 | 1,514 | 92.6 |
| Yes | 4,311 | 5.6 | 3,424 | 5.3 | 887 | 7.1 | 121 | 7.4 |

Note: PMHD: Pre-existing mental health disorder; TWD: New Taiwan Dollar; SD: Standard deviation.
