# Supplementary material for: Disparities in screening participation and stage at diagnosis for breast and bowel cancers among individuals with pre-existing mental health disorders: A population-based study
Source: PLoS One. 2026 Oct 27;21(10):e0361617. doi: 10.1371/journal.pone.0361617 (PMC13712726; doi:10.1371/journal.pone.0361617)
Supplement: Supplementary file 4 [file pone.0361617.s004.docx]

| Variables | Total | | Without PMHD | | With PMHD | | With Severe PMHD | |
| --- | --- | --- | --- | --- | --- | --- | --- | --- |
|  | n | % | n | % | n | % | n | % |
| Total | 79,142 | 100.0 | 65,559 | 100.0 | 13,583 | 100.0 | 2,639 | 100.0 |
| Sex |  |  |  |  |  |  |  |  |
| Female | 34,275 | 43.3 | 27,288 | 41.6 | 6,987 | 51.4 | 1,367 | 51.8 |
| Male | 44,867 | 56.7 | 38,271 | 58.4 | 6,596 | 48.6 | 1,272 | 48.2 |
| Age at diagnosis (years old) |  |  |  |  |  |  |  |  |
| <45 | 4,989 | 6.3 | 4,541 | 6.9 | 448 | 3.3 | 104 | 3.9 |
| 45-54 | 11,053 | 14.0 | 9,761 | 14.9 | 1,292 | 9.5 | 221 | 8.4 |
| 55-64 | 19,488 | 24.6 | 16,865 | 25.7 | 2,623 | 19.3 | 379 | 14.4 |
| 65+ | 43,612 | 55.1 | 34,392 | 52.5 | 9,220 | 67.9 | 1,935 | 73.3 |
| Mean ± SD | 66.7 ± 13.7 | | 65.8 ± 13.5 | | 71.3 ± 13.4 | | 74.1 ± 14.5 | |
| Monthly income (TWD) |  |  |  |  |  |  |  |  |
| Under 22,000 | 21,652 | 27.4 | 17,226 | 26.3 | 4,426 | 32.6 | 1,032 | 39.1 |
| 22,001-24,000 | 26,817 | 33.9 | 22,074 | 33.7 | 4,743 | 34.9 | 918 | 34.8 |
| 24,001-40,100 | 12,996 | 16.4 | 11,167 | 17.0 | 1,829 | 13.5 | 302 | 11.4 |
| Above 40,101 | 17,677 | 22.3 | 15,092 | 23.0 | 2,585 | 19.0 | 387 | 14.7 |
| Urbanization |  |  |  |  |  |  |  |  |
| Level 1 | 21,441 | 27.1 | 17,712 | 27.0 | 3,729 | 27.5 | 645 | 24.4 |
| Level 2 | 25,050 | 31.7 | 20,849 | 31.8 | 4,201 | 30.9 | 813 | 30.8 |
| Level 3 | 13,817 | 17.5 | 11,589 | 17.7 | 2,228 | 16.4 | 444 | 16.8 |
| Level 4 | 10,856 | 13.7 | 8,899 | 13.6 | 1,957 | 14.4 | 424 | 16.1 |
| Level 5 | 1,743 | 2.2 | 1,427 | 2.2 | 316 | 2.3 | 84 | 3.2 |
| Level 6 | 3,065 | 3.9 | 2,453 | 3.7 | 612 | 4.5 | 144 | 5.5 |
| Level 7 | 3,170 | 4.0 | 2,630 | 4.0 | 540 | 4.0 | 85 | 3.2 |
| Charlson Comorbidity Score |  |  |  |  |  |  |  |  |
| 0 | 25,602 | 32.4 | 22,771 | 34.7 | 2,831 | 20.8 | 327 | 12.4 |
| 1 | 22,804 | 28.8 | 19,310 | 29.5 | 3,494 | 25.7 | 567 | 21.5 |
| Above 2 | 30,736 | 38.8 | 23,478 | 35.8 | 7,258 | 53.4 | 1,745 | 66.1 |
| Cancer history |  |  |  |  |  |  |  |  |
| No | 72,823 | 92.0 | 60,619 | 92.5 | 12,204 | 89.9 | 2,382 | 90.3 |
| Yes | 6,319 | 8.0 | 4,940 | 7.5 | 1,379 | 10.2 | 257 | 9.7 |

Note: PMHD: Pre-existing mental health disorder; TWD: New Taiwan Dollar; SD: Standard deviation.
