# Supplementary material for: Disparities in screening participation and stage at diagnosis for breast and bowel cancers among individuals with pre-existing mental health disorders: A population-based study
Source: PLoS One. 2026 Oct 27;21(10):e0361617. doi: 10.1371/journal.pone.0361617 (PMC13712726; doi:10.1371/journal.pone.0361617)
Supplement: Supplementary file 5 [file pone.0361617.s005.docx]

| Variables | Stage at breast cancer diagnosis | | | | | | | | | |
| --- | --- | --- | --- | --- | --- | --- | --- | --- | --- | --- |
|  | Stages I & II | | Stages III & IV | | p value | aOR | 95% CI | | | p-value |
|  | n | % | n | % |  |  |  | | |  |
| **Breast cancer cohort** |  |  |  |  |  |  |  |  |  |  |
| PMHD |  |  |  |  | <0.001 |  |  |  |  |  |
| Without (ref.) | 50,021 | 77.03 | 14,916 | 22.97 |  | 1 |  |  |  |  |
| With PMHD | 9,902 | 79.34 | 2578 | 20.66 |  | 0.80 | 0.76 | - | 0.83 | <0.001 |
| With severe PMHD | 1,222 | - | 413 | - |  | 0.98 | 0.88 | - | 1.11 | 0.805 |
| **Bowel cancer cohort** |  |  |  |  |  |  |  |  |  |  |
| PMHD |  |  |  |  | <0.001 |  |  |  |  |  |
| Without (ref.) | 27807 | 42.42 | 37752 | 57.58 |  | 1 |  |  |  |  |
| With PMHD | 6062 | 44.63 | 7521 | 55.37 |  | 0.93 | 0.90 | - | 0.97 | <0.001 |
| With severe PMHD | 1112 | - | 1527 | - |  | 1.03 | 0.95 | - | 1.12 | 0.440 |

Note:

1. aOR, adjusted odds ratio. Adjustments contained sex, age, monthly income, urbanization, Charlson comorbidity score, and cancer history.

2. PMHD: Pre-existing mental health disorder
