# Supplementary material for: Disparities in screening participation and stage at diagnosis for breast and bowel cancers among individuals with pre-existing mental health disorders: A population-based study
Source: PLoS One. 2026 Oct 27;21(10):e0361617. doi: 10.1371/journal.pone.0361617 (PMC13712726; doi:10.1371/journal.pone.0361617)
Supplement: Supplementary file 6 [file pone.0361617.s006.docx]

| Variables | Total | | Without PMHD | | With PMHD | | With Severe PMHD | |
| --- | --- | --- | --- | --- | --- | --- | --- | --- |
|  | n | % | n | % | n | % | n | % |
| Total | 1,534,698 | 100.0 | 1,278,915 | 100.0 | 255,783 | 100.0 | 22,036 | 8.6 |
| Age (years) |  |  |  |  |  |  |  |  |
| 45-49 | 264,552 | 17.2 | 220,460 | 17.2 | 44,092 | 17.2 | 4,023 | 18.3 |
| 50-54 | 306,114 | 20.0 | 255,095 | 20.0 | 51,019 | 20.0 | 4,366 | 19.8 |
| 55-59 | 339,528 | 22.1 | 282,940 | 22.1 | 56,588 | 22.1 | 4,694 | 21.3 |
| 60-64 | 319,554 | 20.8 | 266,295 | 20.8 | 53,259 | 20.8 | 4,359 | 19.8 |
| 64-69 | 304,950 | 19.9 | 254,125 | 19.9 | 50,825 | 19.9 | 4,594 | 20.9 |
| Mean ± SD | 57.3 ± 7.0 | | 57.3 ± 7.0 | | 57.3 ± 7.0 | | 57.3 ± 7.1 | |
| Monthly income (TWD) |  |  |  |  |  |  |  |  |
| Under 23,100 | 418,806 | 27.3 | 349,005 | 27.3 | 69,801 | 27.3 | 7,498 | 34.0 |
| 23,101-24,000 | 433,974 | 28.3 | 361,645 | 28.3 | 72,329 | 28.3 | 6,281 | 28.5 |
| 24,001-40,100 | 340,470 | 22.2 | 283,725 | 22.2 | 56,745 | 22.2 | 4,266 | 19.4 |
| Above 40,100 | 341,448 | 22.3 | 284,540 | 22.3 | 56,908 | 22.3 | 3,991 | 18.1 |
| Urbanization |  |  |  |  |  |  |  |  |
| Level 1 | 461,885 | 30.1 | 388,064 | 30.3 | 73,821 | 28.9 | 5,754 | 26.1 |
| Level 2 | 539,390 | 35.2 | 451,293 | 35.3 | 88,097 | 34.4 | 7,390 | 33.5 |
| Level 3 | 243,730 | 15.9 | 201,642 | 15.8 | 42,088 | 16.5 | 3,752 | 17.0 |
| Level 4 | 179,284 | 11.7 | 147,426 | 11.5 | 31,858 | 12.5 | 3,029 | 13.8 |
| Level 5 | 20,634 | 1.3 | 16,928 | 1.3 | 3,706 | 1.5 | 370 | 1.7 |
| Level 6 | 43,217 | 2.8 | 35,246 | 2.8 | 7,971 | 3.1 | 883 | 4.0 |
| Level 7 | 46,558 | 3.0 | 38,316 | 3.0 | 8,242 | 3.2 | 858 | 3.9 |
| Charlson Comorbidity Score |  |  |  |  |  |  |  |  |
| 0 | 957,811 | 62.4 | 849,447 | 66.4 | 108,364 | 42.4 | 7,657 | 34.8 |
| 1 | 329,296 | 21.5 | 254,135 | 19.9 | 75,161 | 29.4 | 6,001 | 27.2 |
| Above 2 | 247,591 | 16.1 | 175,333 | 13.7 | 72,258 | 28.3 | 8,378 | 38.0 |
| Average annual outpatient visits |  |  |  |  |  |  |  |  |
| <Q1 (Low) | 396,837 | 25.9 | 388,303 | 30.4 | 8,534 | 3.3 | 955 | 4.3 |
| Q1~Q3 (Medium) | 746,400 | 48.6 | 638,333 | 49.9 | 108,067 | 42.3 | 7,470 | 33.9 |
| >Q3 (High) | 391,461 | 25.5 | 252,279 | 19.7 | 139,182 | 54.4 | 13,611 | 61.8 |

Note:

1. Q1 = 4, Q2 = 11, Q3 = 21.

2. PMHD: Pre-existing mental health disorder; TWD: New Taiwan Dollar; SD: Standard deviation.
