# Supplementary material for: Disparities in screening participation and stage at diagnosis for breast and bowel cancers among individuals with pre-existing mental health disorders: A population-based study
Source: PLoS One. 2026 Oct 27;21(10):e0361617. doi: 10.1371/journal.pone.0361617 (PMC13712726; doi:10.1371/journal.pone.0361617)
Supplement: Supplementary file 7 [file pone.0361617.s007.docx]

| Variables | Total | | Without PMHD | | With PMHD | | With severe PMHD | |
| --- | --- | --- | --- | --- | --- | --- | --- | --- |
|  | n | % | n | % | n | % | n | % |
| Total | 2,682,756 | 100.0 | 2,235,630 | 100.0 | 447,126 | 100.0 | 42,319 | 9.5 |
| Sex |  |  |  |  |  |  |  |  |
| Male | 1,213,548 | 45.2 | 1,011,290 | 45.2 | 202,258 | 45.2 | 20,957 | 49.5 |
| Female | 1,469,208 | 54.8 | 1,224,340 | 54.8 | 244,868 | 54.8 | 21,362 | 50.5 |
| Age (years) |  |  |  |  |  |  |  |  |
| 50-54 | 595,038 | 22.2 | 495,865 | 22.2 | 99,173 | 22.2 | 9,648 | 22.8 |
| 55-59 | 631,626 | 23.5 | 526,355 | 23.5 | 105,271 | 23.5 | 9,636 | 22.8 |
| 60-64 | 579,816 | 21.6 | 483,180 | 21.6 | 96,636 | 21.6 | 8,539 | 20.2 |
| 64-69 | 537,480 | 20.0 | 447,900 | 20.0 | 89,580 | 20.0 | 8,500 | 20.1 |
| 70-74 | 338,796 | 12.6 | 282,330 | 12.6 | 56,466 | 12.6 | 5,996 | 14.2 |
| Mean ± SD | 60.8 ± 6.7 | | 60.8 ± 6.7 | | 60.8 ± 6.7 | | 61.0 ± 6.9 | |
| Monthly income (TWD) |  |  |  |  |  |  |  |  |
| Under 23,100 | 769,128 | 28.7 | 640,940 | 28.7 | 128,188 | 28.7 | 15,499 | 36.6 |
| 23,101-24,000 | 755,220 | 28.2 | 629,350 | 28.2 | 125,870 | 28.2 | 12,076 | 28.5 |
| 24,001-40,100 | 531,960 | 19.8 | 443,300 | 19.8 | 88,660 | 19.8 | 7,322 | 17.3 |
| Above 40,100 | 626,448 | 23.4 | 522,040 | 23.4 | 104,408 | 23.4 | 7,422 | 17.5 |
| Urbanization |  |  |  |  |  |  |  |  |
| Level 1 | 762,470 | 28.4 | 639,644 | 28.6 | 122,826 | 27.5 | 10,441 | 24.7 |
| Level 2 | 914,826 | 34.1 | 765,990 | 34.3 | 148,836 | 33.3 | 13,584 | 32.1 |
| Level 3 | 441,487 | 16.5 | 365,235 | 16.3 | 76,252 | 17.1 | 7,193 | 17.0 |
| Level 4 | 336,221 | 12.5 | 277,793 | 12.4 | 58,428 | 13.1 | 6,246 | 14.8 |
| Level 5 | 44,023 | 1.6 | 36,259 | 1.6 | 7,764 | 1.7 | 945 | 2.2 |
| Level 6 | 91,825 | 3.4 | 75,177 | 3.4 | 16,648 | 3.7 | 2,024 | 4.8 |
| Level 7 | 91,904 | 3.4 | 75,532 | 3.4 | 16,372 | 3.7 | 1,886 | 4.5 |
| Charlson Comorbidity Score |  |  |  |  |  |  |  |  |
| 0 | 1,703,084 | 63.5 | 1,500,293 | 67.1 | 202,791 | 45.4 | 15,453 | 36.5 |
| 1 | 547,555 | 20.4 | 423,266 | 18.9 | 124,289 | 27.8 | 11,338 | 26.8 |
| Above 2 | 432,117 | 16.1 | 312,071 | 14.0 | 120,046 | 26.9 | 15,528 | 36.7 |
| Average annual outpatient visits |  |  |  |  |  |  |  |  |
| <Q1 (Low) | 736,936 | 27.5 | 718,507 | 32.1 | 18,429 | 4.1 | 2,217 | 5.2 |
| Q1~Q3 (Medium) | 1,200,432 | 44.8 | 1,020,411 | 45.6 | 180,021 | 40.3 | 14,360 | 33.9 |
| >Q3 (High) | 745,388 | 27.8 | 496,712 | 22.2 | 248,676 | 55.6 | 25,742 | 60.8 |

Note:

1. Q1 = 4, Q2 = 12, Q3 = 23.

2. PMHD: Pre-existing mental health disorder; TWD: New Taiwan Dollar; SD: Standard deviation.
