# Supplementary material for: Disparities in screening participation and stage at diagnosis for breast and bowel cancers among individuals with pre-existing mental health disorders: A population-based study
Source: PLoS One. 2026 Oct 27;21(10):e0361617. doi: 10.1371/journal.pone.0361617 (PMC13712726; doi:10.1371/journal.pone.0361617)
Supplement: Supplementary file 8 [file pone.0361617.s008.docx]

| Comparison | Stage 0, n (%) | Stage Unknown, n (%) | Total | χ2 | P-value |
| --- | --- | --- | --- | --- | --- |
| *Breast cancer* |  |  |  |  |  |
| Non-PMHD | 12,139 (74.8%) | 4,086 (25.2%) | 16,225 | - | - |
| Overall PMHD | 2,272 (77.5%) | 661 (22.5%) | 2,933 | 9.19 | 0.002 |
| Severe PMHD | 276 (76.0%) | 87 (24.0%) | 363 | 0.22 | 0.640 |
|  |  |  |  |  |  |
| *Bowel cancer* |  |  |  |  |  |
| Non-PMHD | 7,840 (32.2%) | 16,505 (67.8%) | 24,345 | - | - |
| Overall PMHD | 1,835 (31.3%) | 4,033 (68.7%) | 5,868 | 1.85 | 0.174 |
| Severe PMHD | 312 (28.0%) | 803 (72.0%) | 1,115 | 8.54 | 0.004 |

Note: PMHD: pre-existing mental health disorder
