# Supplementary material for: Disparities in screening participation and stage at diagnosis for breast and bowel cancers among individuals with pre-existing mental health disorders: A population-based study
Source: PLoS One. 2026 Oct 27;21(10):e0361617. doi: 10.1371/journal.pone.0361617 (PMC13712726; doi:10.1371/journal.pone.0361617)
Supplement: Supplementary file 10 [file pone.0361617.s010.docx]

2016-2021 New-onset breast cancer patients (N = 97,806)

Women with breast cancer for analysis

(N = 77,417)

Exclusion:

(1) Age under 18 (n = 11)
(2) Male (n = 387)
(3) Missing information on sex, income, or urbanization (n = 833)
(4) Missing information on stage or carcinoma in situ (n = 19,158)
