# Supplementary material for: Disparities in screening participation and stage at diagnosis for breast and bowel cancers among individuals with pre-existing mental health disorders: A population-based study
Source: PLoS One. 2026 Oct 27;21(10):e0361617. doi: 10.1371/journal.pone.0361617 (PMC13712726; doi:10.1371/journal.pone.0361617)
Supplement: Supplementary file 11 [file pone.0361617.s011.docx]

2016-2021 New-onset bowel cancer patients (N = 110,253)

People with bowel cancer for analysis

(N = 79,142)

Exclusion:

(1) Age under 18 (n = 34)
(2) Missing information on sex, income, or urbanization (n = 864)
(3) Missing information on stage or carcinoma in situ (n = 30,213)
