# Supplementary material for: Disparities in screening participation and stage at diagnosis for breast and bowel cancers among individuals with pre-existing mental health disorders: A population-based study
Source: PLoS One. 2026 Oct 27;21(10):e0361617. doi: 10.1371/journal.pone.0361617 (PMC13712726; doi:10.1371/journal.pone.0361617)
Supplement: Supplementary file 12 [file pone.0361617.s012.docx]

2016

2019

2021

*People who were eligible for screening*

*History of mental health disorders*

Observation:

Cancer screening attendance

2018
