# Supplementary material for: Disparities in screening participation and stage at diagnosis for breast and bowel cancers among individuals with pre-existing mental health disorders: A population-based study
Source: PLoS One. 2026 Oct 27;21(10):e0361617. doi: 10.1371/journal.pone.0361617 (PMC13712726; doi:10.1371/journal.pone.0361617)
Supplement: Supplementary file 13 [file pone.0361617.s013.docx]

Women age 45-69 in 2019

(N = 4,525,214)

Women age 45-69

(N = 4,126,215)

Exclusion:

(1) Patients diagnosed with cancer before 2019 (n =291,712)
(2) Patients who suffered from cancer from 2019 to 2021 (n =83,224)
(3) Patients who died during 2016-2019 (n =24,063)

Without PMHD

(N = 3,870,432)

With PMHD

(N = 255,783)

Without PMHD

(N = 2,774,445)

Excluded women diagnosed with any mental health disease code between 2016 and 2021 (n = 1,095,987)

Without PMHD

(N =1,278,915)

With PMHD

(N =255,783)

Matched via propensity score matching in 1: 5 scales based on age and monthly income.
