# Supplementary material for: Disparities in screening participation and stage at diagnosis for breast and bowel cancers among individuals with pre-existing mental health disorders: A population-based study
Source: PLoS One. 2026 Oct 27;21(10):e0361617. doi: 10.1371/journal.pone.0361617 (PMC13712726; doi:10.1371/journal.pone.0361617)
Supplement: Supplementary file 14 [file pone.0361617.s014.docx]

Patients age 50-74 in 2019

(N = 8,092,821)

Patients age 50-74

(N =7,202,662)

Exclusion:

(1) Patients diagnosed with cancer before 2019 (n = 602,430)
(2) Patients who suffered from cancer from 2019 to 2021 (n = 181,241)
(3) Patients who died during 2016-2019 (n = 106,488)

Without PMHD

(N =6,755,536)

With PMHD

(N =447,126)

Without PMHD

(N = 5,006,502)

Excluded people diagnosed with any mental health disease code between 2016 and 2021 (n =1,749,034)

Without PMHD

(N = 2,235,630)

With PMHD

(N = 447,126)

Matched via propensity score matching in 1: 5 scales based on sex, age and monthly income.
